# Editorial: Insights in microorganisms in vertebrate digestive systems: 2023

**DOI:** 10.3389/fmicb.2025.1588178

**Published:** 2025-04-08

**Authors:** Thi Thu Hao Van, Klibs N. Galvao

**Affiliations:** ^1^School of Science, RMIT University, Bundoora, VIC, Australia; ^2^Department of Large Animal Clinical Sciences, University of Florida, Gainesville, FL, United States

**Keywords:** microbiota, gut, health, disease, vertebrate

The digestive system of vertebrates hosts a diverse array of microorganisms that play vital roles in nutrition, immune function, and overall health. As research progresses, the multifaceted relationships between gut microorganisms and their hosts are increasingly recognized for their potential to influence health outcomes and disease processes. This Research Topic focuses on the complex interactions between gut microbiota and vertebrate health, highlighting recent studies that reveal their impact on health conditions and developmental processes. This Research Topic provides valuable insights into how these microbial communities can be leveraged for disease management and health optimization.

The study by Yang M. et al. investigated the complex interactions between gut microbiota and endocarditis using a bidirectional Mendelian randomization approach. The authors identified protective microbial taxa, such as Victivallaceae, *Eubacterium fissicatena, Escherichia/Shigella, Peptococcus*, and *Sellimonas*, while *Blautia* and *Ruminococcus* were found to be associated with an increased risk. This research highlights the therapeutic potential of targeting the gut microbiota to reduce the risk of cardiovascular disease.

In ruminant nutrition, research by Ma et al. highlighted how dietary energy levels impact rumen microbiota and metabolites in yaks. The authors revealed how varying nutritional energy inputs can reshape microbial communities and fermentation parameters, which provides a scientific basis for optimizing yak diets. Similarly, Liu et al. investigated the impact of crude protein content in Huanjiang mini pigs, showing the importance of balanced protein levels for intestinal health and growth performance.

Examining the gut microbiota during weaning, Yang Q. et al. showed significant shifts in microbial composition and serum metabolites in Dezhou donkey foals. Their work highlights essential adaptations for the transition from milk to solid feed, offering insights into effective nutritional strategies for young mammals.

The study by Li et al. on *Eimeria* infections in plateau pikas examined the effects on their gut microbiome, behavior, and physiology. They found that while overall bacterial community structures were stable, *Eimeria* influenced temporal variation, notably affecting the genus *Ruminococcus*. Changes in thyroid hormones and exploratory behavior suggest a potential energy trade-off. From a different angle, Xu C. et al. focused on *Christensenella minuta*, exploring its interactions within the gut microbiota network. *C. minuta* was found to support beneficial bacteria, enhancing overall microbial diversity and stability. This research highlights its potential role in promoting gastrointestinal health and microbiome management strategies.

The study by Fagundes et al. explored how *Faecalibacterium prausnitzii* may reduce inflammation in inflammatory bowel disease through the HIF1α pathway. Their findings suggest that these bacteria aid in managing IBD by promoting healing and offering new treatment possibilities. This was complemented by research from Meng et al. on the Traditional Chinese Medicine Xielikang, which modulates the gut microbiota in AIDS patients to reduce diarrhea symptoms, adding a complementary approach to managing gut health and immunity.

Other significant studies include work by Gao, Wang et al., who linked gut microbiota to juvenile idiopathic arthritis through Mendelian randomization, identifying plasma metabolites as potential mediators. The research by Yang X. et al. associated sheep fecal scores with gastrointestinal microorganisms, indicating specific bacteria that influence growth and health indices in livestock. The study by Gao Q. et al. examined the impact of dietary D-lactate on rumen fermentation in beef cattle. Higher levels of D-lactate increased gas and volatile fatty acid production and altered rumen pH and bacterial communities. While improving energy availability, it also raised methane emissions and the prevalence of *Escherichia-Shigella*. The research by Ding et al. on Hong-bailanshen supplementation in horses highlighted improvements in antioxidant enzyme activity and beneficial gut bacteria, suggesting dietary interventions for improved equine health.

Research by Xu Y. et al. revealed age-related shifts in buffalo rumen microbiota, highlighting their role in lignocellulose degradation and immune response in adults, and galactose conversion and antibiotic synthesis in breastfed calves. These insights deepen our understanding of how gut microorganisms adapt to different diets and growth stages. Additionally, Wang et al. uncovered distinct microbiome signatures in hemorrhoids compared to surrounding areas, with *Staphylococcus* prevalent on surfaces and Prevotella associated with thrombosed hemorrhoids. These findings suggest a microbial role in hemorrhoid development. Finally, Gao, Zhou et al. investigated the causal link between *Lachnospiraceae* abundance and appendicular lean mass (ALM) using Mendelian randomization. Their study found that increased *Lachnospiraceae* is associated with higher ALM, with Aminopeptidase O Protein (AOPEP) as a mediator. The findings indicate that targeting gut microbiota and AOPEP could improve sarcopenia management in elderly patients.

Taken together, these studies illuminate how gut microbiota specifically affect vertebrate health, offering promising avenues for advancing health and disease management through microbial strategies. Continued research in this field could lead to innovative treatments and preventative measures for a variety of health conditions.

